# Supplementary material for: Interpretable and Calibrated Classification of Clinical Data Using Supervised Feature Binarization
Source: Bioengineering (Basel). 2026 Oct 2;13(10):1152. doi: 10.3390/bioengineering13101152 (PMC13701737; doi:10.3390/bioengineering13101152)
Supplement: Supplementary file 1 [file bioengineering-13-01152-s001.zip › bioengineering-4599064-supplementary.pdf]

Supplementary Materials

Interpretable and Calibrated Classification of Clinical Data Using Supervised Feature Binarization

Antony Garcia, Adrián Noriega de la Colina, Gabrielle Britton and Xinming Huang
Correspondence: xhuang@wpi.edu · Code and data: https://github.com/AntonyGarcia/chi2-binarized-naive-bayes

Contents

Table S1 Complete comparison for the Pima Indians Diabetes Database (Table 6 of the main text) p. 2
Table S2 Complete comparison for the Wisconsin Breast Cancer dataset (Table 7 of the main text) p. 4
Table S3 Complete comparison for the Heart Failure Prediction dataset (Table 8 of the main text) p. 5

Notes on Tables S1–S3

- **Evaluation protocol.** All models, including the re-implementations of the comparison studies, are evaluated with stratified 10-fold cross-validation (seed 42). Every metric is computed once on the pooled out-of-fold predictions, and the value in brackets is its 95% bootstrap confidence interval (1000 replicates).
- **Metrics.** Precision, recall and F1-score are weighted averages over the two classes; AUC is the area under the receiver operating characteristic curve.
- **Statistical tests.** DeLong's test (AUC) and McNemar's test (paired predictions) compare each model with the Bernoulli Naïve Bayes (BNB) reference of its block, shown in grey. p-values are adjusted with the Benjamini–Hochberg procedure within each table and test, as in the main text. Bold p-values are significant ($p < 0.05$); * denotes significant outperformance over BNB and ** significant underperformance.
- **Blocks.** Each shaded row names the comparison study and, for the Pima Indians Diabetes Database, the preprocessing of that study. The re-implementations follow the model and preprocessing descriptions of each study, so their values are not expected to match the published ones exactly.
- **Reproducibility.** All values are produced by the code in the repository listed above.

Table S1. Complete comparison for the Pima Indians Diabetes Database (extends Table 6 of the main text). In the blocks that include a NaN-aware model, the NaN-aware BNB is the reference.

**Algorithm**	**Accuracy**	**Precision**	**Recall**	**F1-score**	**AUC**	**DeLong**	**McNemar**
	value and 95% confidence interval					adjusted p-value	
**Ashour et al. (2024)** [62] *Mean imputation*							
Gaussian Naïve Bayes	0.760 [0.729, 0.789]	0.757 [0.726, 0.786]	0.760 [0.729, 0.789]	0.758 [0.726, 0.787]	0.820 [0.790, 0.849]	**0.022***	0.599
k-Nearest Neighbors	0.732 [0.699, 0.763]	0.729 [0.696, 0.762]	0.732 [0.699, 0.763]	0.730 [0.696, 0.761]	0.779 [0.744, 0.811]	0.400	0.600
J48 Decision Tree	0.725 [0.690, 0.755]	0.722 [0.686, 0.753]	0.725 [0.690, 0.755]	0.724 [0.687, 0.754]	0.707 [0.670, 0.740]	**<0.001****	0.505
Random Forest	0.757 [0.725, 0.785]	0.752 [0.721, 0.783]	0.757 [0.725, 0.785]	0.754 [0.722, 0.783]	0.825 [0.795, 0.854]	**0.010***	0.773
Feedforward Neural Network	0.767 [0.737, 0.796]	0.761 [0.730, 0.792]	0.767 [0.737, 0.796]	0.759 [0.727, 0.789]	0.835 [0.806, 0.862]	**<0.001***	0.505
Convolutional Neural Network	0.779 [0.749, 0.807]	0.775 [0.745, 0.804]	0.779 [0.749, 0.807]	0.775 [0.745, 0.804]	0.835 [0.806, 0.863]	**<0.001***	0.196
Bernoulli Naïve Bayes	0.732 [0.702, 0.763]	0.730 [0.699, 0.761]	0.732 [0.702, 0.763]	0.731 [0.700, 0.762]	0.784 [0.751, 0.815]	0.116	0.245
Bernoulli Naïve Bayes (NaN-aware) (reference)	0.747 [0.716, 0.775]	0.744 [0.712, 0.773]	0.747 [0.716, 0.775]	0.745 [0.713, 0.774]	0.793 [0.759, 0.823]	–	–
**Battineni et al. (2019)** [63] *Removal of missing values*							
Gaussian Naïve Bayes	0.770 [0.730, 0.814]	0.771 [0.731, 0.816]	0.770 [0.730, 0.814]	0.771 [0.731, 0.814]	0.831 [0.790, 0.872]	0.792	0.600
J48 Decision Tree	0.791 [0.750, 0.829]	0.806 [0.767, 0.845]	0.791 [0.750, 0.829]	0.795 [0.756, 0.834]	0.801 [0.750, 0.849]	0.302	1.000
Random Forest	0.788 [0.745, 0.832]	0.784 [0.740, 0.829]	0.788 [0.745, 0.832]	0.785 [0.741, 0.829]	0.855 [0.813, 0.896]	0.127	0.924
Logistic Regression	0.781 [0.737, 0.819]	0.774 [0.729, 0.817]	0.781 [0.737, 0.819]	0.773 [0.728, 0.814]	0.849 [0.808, 0.890]	0.246	0.924
Bernoulli Naïve Bayes (reference)	0.788 [0.747, 0.829]	0.791 [0.752, 0.834]	0.788 [0.747, 0.829]	0.790 [0.749, 0.830]	0.827 [0.783, 0.869]	–	–
**Bhoi et al. (2021)** [64] *Removal of missing values*							
Classification Tree	0.730 [0.686, 0.776]	0.732 [0.688, 0.778]	0.730 [0.686, 0.776]	0.731 [0.687, 0.776]	0.699 [0.649, 0.748]	**<0.001****	0.176
Support Vector Machine	0.760 [0.719, 0.804]	0.753 [0.710, 0.799]	0.760 [0.719, 0.804]	0.753 [0.709, 0.798]	0.836 [0.790, 0.879]	0.652	0.505
k-Nearest Neighbors	0.763 [0.719, 0.804]	0.756 [0.711, 0.801]	0.763 [0.719, 0.804]	0.758 [0.712, 0.801]	0.797 [0.750, 0.845]	0.237	0.599
Gaussian Naïve Bayes	0.770 [0.730, 0.814]	0.771 [0.731, 0.816]	0.770 [0.730, 0.814]	0.771 [0.731, 0.814]	0.831 [0.790, 0.872]	0.792	0.600
Random Forest	0.783 [0.742, 0.829]	0.780 [0.739, 0.826]	0.783 [0.742, 0.829]	0.781 [0.739, 0.827]	0.851 [0.810, 0.892]	0.158	0.924
Multilayer Perceptron	0.755 [0.712, 0.798]	0.755 [0.710, 0.801]	0.755 [0.712, 0.798]	0.755 [0.711, 0.799]	0.804 [0.752, 0.851]	0.314	0.505
AdaBoost	0.781 [0.737, 0.819]	0.775 [0.731, 0.817]	0.781 [0.737, 0.819]	0.776 [0.731, 0.817]	0.843 [0.798, 0.886]	0.302	0.924
Logistic Regression	0.783 [0.742, 0.821]	0.777 [0.732, 0.819]	0.783 [0.742, 0.821]	0.776 [0.733, 0.818]	0.849 [0.808, 0.889]	0.240	0.924
Bernoulli Naïve Bayes (reference)	0.788 [0.747, 0.829]	0.791 [0.752, 0.834]	0.788 [0.747, 0.829]	0.790 [0.749, 0.830]	0.827 [0.783, 0.869]	–	–
**Pranto et al. (2020)** [65] *Mean imputation, feature selection*							
Gaussian Naïve Bayes	0.758 [0.728, 0.786]	0.753 [0.721, 0.783]	0.758 [0.728, 0.786]	0.754 [0.722, 0.783]	0.829 [0.799, 0.857]	**0.002***	0.196
Decision Tree	0.745 [0.712, 0.775]	0.737 [0.704, 0.768]	0.745 [0.712, 0.775]	0.734 [0.699, 0.767]	0.759 [0.725, 0.790]	**0.047****	0.599
Random Forest	0.755 [0.725, 0.785]	0.752 [0.720, 0.783]	0.755 [0.725, 0.785]	0.753 [0.722, 0.783]	0.812 [0.779, 0.842]	0.208	0.388
k-Nearest Neighbors	0.772 [0.741, 0.802]	0.767 [0.734, 0.799]	0.772 [0.741, 0.802]	0.766 [0.734, 0.798]	0.836 [0.806, 0.863]	**0.002***	0.100
Bernoulli Naïve Bayes	0.717 [0.684, 0.747]	0.709 [0.676, 0.741]	0.717 [0.684, 0.747]	0.711 [0.678, 0.742]	0.788 [0.755, 0.818]	0.252	0.505
Bernoulli Naïve Bayes (NaN-aware) (reference)	0.728 [0.694, 0.757]	0.720 [0.684, 0.751]	0.728 [0.694, 0.757]	0.721 [0.685, 0.752]	0.793 [0.758, 0.823]	–	–

Table S1. Complete comparison for the Pima Indians Diabetes Database (continued).

Algorithm	Accuracy	Precision	Recall	F1-score	AUC	DeLong	McNemar
	value and 95% confidence interval					adjusted p-value	
**Reza et al. (2023)** [66] *SMOTE, Mean imputation and Outlier Removal*							
SVM (Linear)	0.727 [0.694, 0.757]	0.750 [0.720, 0.780]	0.727 [0.694, 0.757]	0.732 [0.701, 0.761]	0.824 [0.796, 0.853]	**0.019***	0.741
SVM (Polynomial)	0.720 [0.686, 0.753]	0.718 [0.685, 0.751]	0.720 [0.686, 0.753]	0.719 [0.685, 0.752]	0.774 [0.736, 0.810]	0.357	0.599
SVM (RBF Minkowski)	0.704 [0.671, 0.737]	0.695 [0.660, 0.730]	0.704 [0.671, 0.737]	0.698 [0.663, 0.732]	0.735 [0.695, 0.770]	**0.004****	0.388
SVM (RBF City Block)	0.698 [0.664, 0.732]	0.684 [0.647, 0.721]	0.698 [0.664, 0.732]	0.667 [0.628, 0.704]	0.733 [0.695, 0.767]	**0.004****	0.294
SVM (RBF Mahalanobis)	0.742 [0.708, 0.772]	0.737 [0.702, 0.769]	0.742 [0.708, 0.772]	0.724 [0.686, 0.757]	0.788 [0.752, 0.821]	0.761	0.924
SVM (RBF Bray-Curtis)	0.717 [0.686, 0.746]	0.741 [0.708, 0.770]	0.717 [0.686, 0.746]	0.723 [0.692, 0.751]	0.814 [0.784, 0.842]	0.158	0.505
SVM (RBF Canberra)	0.757 [0.727, 0.788]	0.780 [0.752, 0.809]	0.757 [0.727, 0.788]	0.762 [0.733, 0.793]	0.834 [0.805, 0.861]	**<0.001***	0.505
SVM (RBF Euclidean Distance)	0.745 [0.715, 0.776]	0.738 [0.706, 0.770]	0.745 [0.715, 0.776]	0.738 [0.705, 0.770]	0.826 [0.796, 0.856]	**0.019***	0.864
Bernoulli Naïve Bayes	0.699 [0.667, 0.730]	0.724 [0.693, 0.756]	0.699 [0.667, 0.730]	0.705 [0.674, 0.735]	0.780 [0.748, 0.811]	0.252	0.176
Bernoulli Naïve Bayes (NaN-aware) (reference)	0.737 [0.706, 0.767]	0.740 [0.708, 0.771]	0.737 [0.706, 0.767]	0.738 [0.707, 0.768]	0.794 [0.760, 0.826]	–	–

Table S2. Complete comparison for the Wisconsin Breast Cancer dataset (extends Table 7 of the main text).

Algorithm	Accuracy	Precision	Recall	F1-score	AUC	DeLong	McNemar
	value and 95% confidence interval					adjusted p-value	
**Azar and El-Said (2013)** [71]							
Multi-layer Perceptron (MLP)	0.967 [0.953, 0.981]	0.967 [0.952, 0.981]	0.967 [0.953, 0.981]	0.967 [0.952, 0.981]	0.990 [0.978, 0.997]	0.277	0.063
Radial Basis Function Network (RBF)	0.944 [0.924, 0.961]	0.944 [0.925, 0.962]	0.944 [0.924, 0.961]	0.943 [0.923, 0.961]	0.989 [0.980, 0.995]	0.159	1.000
Probabilistic Neural Network (PNN)	0.958 [0.942, 0.974]	0.960 [0.945, 0.975]	0.958 [0.942, 0.974]	0.957 [0.941, 0.973]	0.993 [0.986, 0.998]	**0.029***	0.214
Bernoulli Naïve Bayes (reference)	0.944 [0.924, 0.963]	0.944 [0.924, 0.963]	0.944 [0.924, 0.963]	0.944 [0.924, 0.963]	0.984 [0.974, 0.992]	–	–
**Jakhar et al. (2023)** [68]							
SELF Stacking Ensemble	0.968 [0.954, 0.982]	0.968 [0.954, 0.982]	0.968 [0.954, 0.982]	0.968 [0.954, 0.982]	0.992 [0.984, 0.998]	**0.042***	**0.003***
Extra Trees	0.965 [0.951, 0.979]	0.965 [0.951, 0.979]	0.965 [0.951, 0.979]	0.965 [0.951, 0.979]	0.993 [0.986, 0.998]	**0.016***	**0.032***
Random Forest	0.949 [0.931, 0.965]	0.949 [0.932, 0.965]	0.949 [0.931, 0.965]	0.949 [0.931, 0.965]	0.988 [0.978, 0.996]	0.104	0.634
AdaBoost	0.977 [0.963, 0.988]	0.977 [0.963, 0.988]	0.977 [0.963, 0.988]	0.977 [0.963, 0.988]	0.994 [0.989, 0.998]	**0.016***	**0.003***
Gradient Boosting	0.958 [0.940, 0.974]	0.958 [0.942, 0.974]	0.958 [0.940, 0.974]	0.958 [0.940, 0.974]	0.991 [0.983, 0.996]	**0.042***	0.167
K-Nearest Neighbor (k=9)	0.968 [0.954, 0.982]	0.969 [0.954, 0.983]	0.968 [0.954, 0.982]	0.968 [0.954, 0.982]	0.988 [0.978, 0.997]	0.301	**0.035***
Support Vector Machine (RBF)	0.975 [0.963, 0.986]	0.975 [0.963, 0.986]	0.975 [0.963, 0.986]	0.975 [0.963, 0.986]	0.994 [0.987, 0.999]	**0.029***	**0.002***
Multilayer Perceptron (MLP)	0.974 [0.960, 0.986]	0.974 [0.960, 0.986]	0.974 [0.960, 0.986]	0.974 [0.960, 0.986]	0.991 [0.982, 0.998]	0.177	**0.026***
Classification and Regression Tree (CART)	0.914 [0.891, 0.935]	0.914 [0.891, 0.935]	0.914 [0.891, 0.935]	0.914 [0.891, 0.935]	0.907 [0.882, 0.932]	**<0.001****	**0.026****
Stochastic Gradient Descent (Log-loss)	0.974 [0.960, 0.986]	0.974 [0.960, 0.986]	0.974 [0.960, 0.986]	0.974 [0.959, 0.986]	0.995 [0.989, 0.999]	**0.016***	**0.012***
Bernoulli Naïve Bayes (reference)	0.944 [0.924, 0.963]	0.944 [0.924, 0.963]	0.944 [0.924, 0.963]	0.944 [0.924, 0.963]	0.984 [0.974, 0.992]	–	–
**Naji et al. (2021)** [67]							
Support Vector Machine (SVM)	0.975 [0.963, 0.986]	0.975 [0.963, 0.986]	0.975 [0.963, 0.986]	0.975 [0.963, 0.986]	0.994 [0.987, 0.999]	**0.029***	**0.002***
Random Forest	0.956 [0.938, 0.972]	0.956 [0.939, 0.972]	0.956 [0.938, 0.972]	0.956 [0.938, 0.972]	0.989 [0.978, 0.997]	0.104	0.167
Logistic Regression	0.967 [0.953, 0.981]	0.968 [0.954, 0.981]	0.967 [0.953, 0.981]	0.966 [0.952, 0.981]	0.992 [0.985, 0.998]	**0.042***	**0.039***
Decision Tree (C4.5 approximation)	0.935 [0.914, 0.954]	0.935 [0.915, 0.955]	0.935 [0.914, 0.954]	0.935 [0.914, 0.954]	0.932 [0.909, 0.954]	**<0.001****	0.544
K-Nearest Neighbors (KNN)	0.967 [0.951, 0.981]	0.967 [0.952, 0.981]	0.967 [0.951, 0.981]	0.966 [0.951, 0.981]	0.986 [0.975, 0.996]	0.612	**0.035***
Bernoulli Naïve Bayes (reference)	0.944 [0.924, 0.963]	0.944 [0.924, 0.963]	0.944 [0.924, 0.963]	0.944 [0.924, 0.963]	0.984 [0.974, 0.992]	–	–
**Rovshenov and Peker (2022)** [70]							
Artificial Neural Network (ANN)	0.968 [0.954, 0.982]	0.968 [0.954, 0.982]	0.968 [0.954, 0.982]	0.968 [0.954, 0.982]	0.990 [0.982, 0.996]	0.131	**0.035***
Support Vector Machine (SVM, RBF)	0.975 [0.961, 0.988]	0.975 [0.962, 0.988]	0.975 [0.961, 0.988]	0.975 [0.961, 0.988]	0.995 [0.990, 0.999]	**0.016***	**0.002***
Random Forest	0.954 [0.937, 0.972]	0.954 [0.937, 0.972]	0.954 [0.937, 0.972]	0.954 [0.937, 0.972]	0.990 [0.980, 0.997]	0.051	0.252
Bernoulli Naïve Bayes (reference)	0.944 [0.924, 0.963]	0.944 [0.924, 0.963]	0.944 [0.924, 0.963]	0.944 [0.924, 0.963]	0.984 [0.974, 0.992]	–	–
**Shah et al. (2022)** [69]							
Decision Tree (Entropy)	0.935 [0.916, 0.954]	0.935 [0.915, 0.955]	0.935 [0.916, 0.954]	0.935 [0.915, 0.954]	0.970 [0.955, 0.984]	0.057	0.540
Gaussian Naïve Bayes	0.931 [0.910, 0.953]	0.931 [0.910, 0.952]	0.931 [0.910, 0.953]	0.931 [0.910, 0.952]	0.984 [0.975, 0.992]	0.878	0.214
Random Forest	0.958 [0.942, 0.974]	0.958 [0.942, 0.974]	0.958 [0.942, 0.974]	0.958 [0.942, 0.974]	0.989 [0.978, 0.997]	0.081	0.123
Bernoulli Naïve Bayes (reference)	0.944 [0.924, 0.963]	0.944 [0.924, 0.963]	0.944 [0.924, 0.963]	0.944 [0.924, 0.963]	0.984 [0.974, 0.992]	–	–

Table S3. Complete comparison for the Heart Failure Prediction dataset (extends Table 8 of the main text). Categorical predictors are modeled with Categorical Naïve Bayes.

Algorithm	Accuracy	Precision	Recall	F1-score	AUC	DeLong	McNemar
	value and 95% confidence interval					adjusted p-value	
**Kumar Mygapula et al. (2023) [73]**							
SVM	0.865 [0.842, 0.888]	0.866 [0.843, 0.889]	0.865 [0.842, 0.888]	0.864 [0.841, 0.888]	0.921 [0.902, 0.940]	0.868	0.144
Random Forest	0.877 [0.856, 0.899]	0.877 [0.857, 0.900]	0.877 [0.856, 0.899]	0.876 [0.856, 0.899]	0.929 [0.911, 0.947]	0.157	**0.009***
Naïve Bayes	0.853 [0.829, 0.877]	0.853 [0.829, 0.877]	0.853 [0.829, 0.877]	0.853 [0.829, 0.877]	0.911 [0.891, 0.930]	0.170	0.685
Logistic Regression	0.851 [0.825, 0.876]	0.851 [0.825, 0.876]	0.851 [0.825, 0.876]	0.851 [0.824, 0.875]	0.911 [0.889, 0.930]	0.170	0.744
KNN	0.861 [0.837, 0.882]	0.861 [0.837, 0.883]	0.861 [0.837, 0.882]	0.860 [0.836, 0.882]	0.910 [0.887, 0.929]	0.225	0.335
Decision Tree	0.800 [0.773, 0.825]	0.800 [0.774, 0.825]	0.800 [0.773, 0.825]	0.800 [0.774, 0.825]	0.798 [0.771, 0.823]	**<0.001****	**0.009****
Bernoulli Naïve Bayes (reference)	0.845 [0.820, 0.870]	0.845 [0.821, 0.871]	0.845 [0.820, 0.870]	0.845 [0.820, 0.871]	0.920 [0.900, 0.938]	–	–
**Liu et al. (2022) [74]**							
Logistic Regression (LR)	0.854 [0.829, 0.878]	0.854 [0.830, 0.878]	0.854 [0.829, 0.878]	0.854 [0.829, 0.878]	0.911 [0.891, 0.930]	0.157	0.535
Random Forest (RF)	0.867 [0.847, 0.890]	0.868 [0.848, 0.891]	0.867 [0.847, 0.890]	0.867 [0.847, 0.890]	0.928 [0.910, 0.945]	0.170	0.062
Extra Trees (ET)	0.862 [0.840, 0.885]	0.862 [0.840, 0.885]	0.862 [0.840, 0.885]	0.861 [0.839, 0.884]	0.926 [0.908, 0.944]	0.316	0.203
GBDT	0.852 [0.829, 0.876]	0.852 [0.829, 0.877]	0.852 [0.829, 0.876]	0.851 [0.828, 0.876]	0.922 [0.903, 0.940]	0.868	0.744
Multilayer Perceptron (MLP)	0.847 [0.825, 0.869]	0.848 [0.825, 0.870]	0.847 [0.825, 0.869]	0.847 [0.824, 0.869]	0.923 [0.905, 0.940]	0.738	0.956
Stacking Ensemble	0.867 [0.846, 0.890]	0.867 [0.847, 0.891]	0.867 [0.846, 0.890]	0.867 [0.845, 0.889]	0.929 [0.911, 0.946]	0.157	0.061
Bernoulli Naïve Bayes (reference)	0.844 [0.820, 0.868]	0.844 [0.820, 0.869]	0.844 [0.820, 0.868]	0.844 [0.820, 0.868]	0.921 [0.902, 0.938]	–	–
**Muhammad et al. (2023) [72]**							
Logistic Regression	0.845 [0.819, 0.867]	0.845 [0.819, 0.867]	0.845 [0.819, 0.867]	0.845 [0.819, 0.867]	0.912 [0.891, 0.930]	0.291	1.000
Decision Tree	0.800 [0.774, 0.825]	0.801 [0.775, 0.826]	0.800 [0.774, 0.825]	0.800 [0.775, 0.825]	0.799 [0.773, 0.824]	**<0.001****	**0.015****
Random Forest	0.861 [0.839, 0.884]	0.861 [0.839, 0.884]	0.861 [0.839, 0.884]	0.861 [0.838, 0.884]	0.927 [0.908, 0.944]	0.157	0.202
Support Vector Machine (RBF)	0.860 [0.836, 0.882]	0.861 [0.837, 0.883]	0.860 [0.836, 0.882]	0.860 [0.835, 0.882]	0.916 [0.894, 0.935]	0.752	0.203
K Nearest Neighbors	0.843 [0.818, 0.865]	0.842 [0.818, 0.865]	0.843 [0.818, 0.865]	0.842 [0.818, 0.865]	0.909 [0.887, 0.927]	0.171	1.000
Gaussian Naïve Bayes	0.843 [0.819, 0.866]	0.843 [0.820, 0.866]	0.843 [0.819, 0.866]	0.843 [0.819, 0.866]	0.909 [0.889, 0.928]	0.170	1.000
Bernoulli Naïve Bayes (reference)	0.844 [0.818, 0.867]	0.844 [0.819, 0.867]	0.844 [0.818, 0.867]	0.844 [0.819, 0.867]	0.918 [0.900, 0.935]	–	–
**Patidar et al. (2022) [75]**							
Random Forest	0.876 [0.855, 0.898]	0.876 [0.856, 0.898]	0.876 [0.855, 0.898]	0.875 [0.855, 0.898]	0.930 [0.911, 0.947]	0.157	**0.009***
Logistic Regression	0.851 [0.825, 0.875]	0.851 [0.825, 0.875]	0.851 [0.825, 0.875]	0.851 [0.824, 0.874]	0.910 [0.889, 0.929]	0.157	0.744
K-Nearest Neighbours (K=13)	0.854 [0.831, 0.879]	0.854 [0.831, 0.879]	0.854 [0.831, 0.879]	0.854 [0.831, 0.879]	0.920 [0.901, 0.939]	0.908	0.640
Bernoulli Naïve Bayes (reference)	0.845 [0.820, 0.870]	0.845 [0.821, 0.871]	0.845 [0.820, 0.870]	0.845 [0.820, 0.871]	0.920 [0.900, 0.938]	–	–

References

Reference numbers correspond to the reference list of the main text.

[62] Ashour, A.F.; Fouda, M.M.; Fadlullah, Z.M.; Ibrahem, M.I. Optimized Neural Networks for Diabetes Classification Using Pima Indians Diabetes Database. In Proceedings of the 2024 IEEE 3rd International Conference on Computing and Machine Intelligence (ICMI), Mt Pleasant, MI, USA, 13–14 April 2024; pp. 1–7. https://doi.org/10.1109/ICMI60790.2024.10585703.

[63] Battineni, G.; Sagaro, G.G.; Nalini, C.; Amenta, F.; Tayebati, S.K. Comparative Machine-Learning Approach: A Follow-Up Study on Type 2 Diabetes Predictions by Cross-Validation Methods. *Machines* **2019**, *7*, 74. https://doi.org/10.3390/machines7040074.

[64] Bhoi, S.K.; Panda, S.K.; Jena, K.K.; Abhisekh, P.A.; Sahoo, K.S.; Sama, N.U.; Pradhan, S.S.; Sahoo, R.R. Prediction of diabetes in females of pima Indian heritage: A complete supervised learning approach. *Turk. J. Comput. Math. Educ.* **2021**, *12*, 3074–3084.

[65] Pranto, B.; Mehnaz, S.M.; Mahid, E.B.; Sadman, I.M.; Rahman, A.; Momen, S. Evaluating Machine Learning Methods for Predicting Diabetes among Female Patients in Bangladesh. *Information* **2020**, *11*, 374. https://doi.org/10.3390/info11080374.

[66] Reza, M.; Hafsha, U.; Amin, R.; Yasmin, R.; Ruhi, S. Improving SVM performance for type II diabetes prediction with an improved non-linear kernel: Insights from the PIMA dataset. *Comput. Methods Programs Biomed. Update* **2023**, *4*, 100118. https://doi.org/10.1016/j.cmpbup.2023.100118.

[67] Naji, M.A.; Filali, S.E.; Aarika, K.; Benlahmar, E.H.; Abdelouhahid, R.A.; Debauche, O. Machine learning algorithms for breast cancer prediction and diagnosis. *Procedia Comput. Sci.* **2021**, *191*, 487–492. https://doi.org/10.1016/j.procs.2021.07.062.

[68] Jakhar, A.K.; Gupta, A.; Singh, M. SELF: A stacked-based ensemble learning framework for breast cancer classification. *Evol. Intell.* **2023**, *17*, 1341–1356. https://doi.org/10.1007/s12065-023-00824-4.

[69] Shah, A.A.; Malik, H.A.M.; Mohammad, A.; Khan, Y.D.; Alourani, A. Machine learning techniques for identification of carcinogenic mutations, which cause breast adenocarcinoma. *Sci. Rep.* **2022**, *12*, 11738. https://doi.org/10.1038/s41598-022-15533-8.

[70] Rovshenov, A.; Peker, S. Performance Comparison of Different Machine Learning Techniques for Early Prediction of Breast Cancer using Wisconsin Breast Cancer Dataset. In Proceedings of the 2022 3rd International Informatics and Software Engineering Conference (IISEC), Ankara, Turkey, 15–16 December 2022; pp. 1–6. https://doi.org/10.1109/IISEC56263.2022.9998248.

[71] Azar, A.T.; El-Said, S.A. Probabilistic neural network for breast cancer classification. *Neural Comput. Appl.* **2013**, *23*, 1737–1751. https://doi.org/10.1007/s00521-012-1134-8.

[72] Muhammad, G.; Naveed, S.; Nadeem, L.; Mahmood, T.; Khan, A.R.; Amin, Y.; Bahaj, S.A.O. Enhancing Prognosis Accuracy for Ischemic Cardiovascular Disease Using K Nearest Neighbor Algorithm: A Robust Approach. *IEEE Access* **2023**, *11*, 97879–97895. https://doi.org/10.1109/ACCESS.2023.3312046.

[73] Kumar Mygapula, S.P.; Lal Saini, M.; Raj Dheeraj, C.S. Performance Evaluation of Machine Learning Algorithms for Prediction of Cardiac Failure. In Proceedings of the 2023 International Conference on Sustainable Communication Networks and Application (ICSCNA), Theni, India, 15–17 November 2023; pp. 1599–1604. https://doi.org/10.1109/ICSCNA58489.2023.10368606.

[74] Liu, J.; Dong, X.; Zhao, H.; Tian, Y. Predictive Classifier for Cardiovascular Disease Based on Stacking Model Fusion. *Processes* **2022**, *10*, 749. https://doi.org/10.3390/pr10040749.

[75] Patidar, S.; Jain, A.; Gupta, A. Comparative Analysis of Machine Learning Algorithms for Heart Disease Predictions. In Proceedings of the 2022 6th International Conference on Intelligent Computing and Control Systems (ICICCS), Madurai, India, 25–27 May 2022; pp. 1340–1344. https://doi.org/10.1109/ICICCS53718.2022.9788408.
